# Species Richness and Similarity of New Zealand Mayfly Communities (Ephemeroptera) Decline with Increasing Latitude and Altitude

**DOI:** 10.3390/insects15100757

**Published:** 2024-09-29

**Authors:** Stephen R. Pohe, Michael J. Winterbourn, Jon S. Harding

**Affiliations:** 1 School of Biological Sciences, University of Canterbury, Christchurch 8140, New Zealand; pohe.environmental@gmail.com (S.R.P.); jon.harding@canterbury.ac.nz (J.S.H.); 2 Pohe Environmental, Whangarei 0112, New Zealand

**Keywords:** ephemeropterans, biogeography, species diversity, beta diversity, stream ecology, latitudinal gradient

## Abstract

**Simple Summary:**

Understanding the distribution of plants and animals is a major focus of ecologists and is of increasing importance as temperatures rise and the world’s climate becomes more unpredictable. One large-scale pattern that has often been observed is for the number of species in a group of interest to be greater in the tropics than towards the poles. Species also tend to decline in number at higher elevations. We examined the distribution of mayfly species, insects commonly found in streams, at multiple sites throughout New Zealand to see whether the numbers and identities of species were related to latitude and altitude. We found that the number of mayfly species declined from north to south, i.e., towards higher latitudes, and less strongly as elevation increased. Furthermore, the species composition of communities became increasingly different with distance along the latitudinal gradient. Our findings suggest that the species gradient has historical origins, with the north of North Island and the South Island mountains likely to have been important regions of speciation 1.5–0.5 million years ago.

**Abstract:**

The distribution of species in relation to latitude and altitude is of fundamental interest to ecologists and is expected to attain increasing importance as the Earth’s climate continues to change. Species diversity is commonly greater at lower than higher latitudes on a global scale, and the similarity of communities frequently decreases with distance. Nevertheless, reasons for such patterns are not well understood. We investigated species richness and changes in community composition of mayflies (Ephemeroptera) over 13 degrees of latitude at 81 locations throughout New Zealand by light-trapping and the benthic sampling of streams. Mayflies were also sampled along an altitudinal gradient on a prominent inactive volcano in the east of North Island. Sampled streams were predominantly in the native forest, at a wide range of altitudes from sea level to c. 1000 m a. s. l. A total of 47 of the 59 described New Zealand mayflies were recorded during the study, along with five undescribed morphospecies. Species richness declined and the degree of dissimilarity (beta diversity) of mayfly communities increased significantly from north to south but less strongly with increasing altitude. Our results suggest that the southward decline in species richness has historical origins with the north of the country having acted as a major refuge and region of speciation during the Pleistocene. The increasing dissimilarity of the northern and southern communities may reflect an increasingly harsh climate, variable amounts of subsequent southward dispersal of northern species and, in the South Island, the presence of species which may have evolved in the newly uplifted mountains during the Miocene–Pliocene.

## 1. Introduction

The relationships among species diversity, latitude, and altitude are of fundamental interest to ecologists and biogeographers and are expected to undergo significant changes, both globally and in New Zealand, as a result of climate change [1,2]. Species diversity is commonly greater at lower than higher latitudes on a global scale, and the similarity of communities commonly decreases with distance [3,4]. Although the reasons for this common global pattern are not well understood [5], Fine [6] noted that tropical regions are the source of almost all groups of organisms and, in contrast to lands at higher latitudes, their greater extent and climatic stability during the last 10–50 million years have promoted increased speciation and reduced extinction rates. The geographical distribution of stream-dwelling insects was reviewed by Vinson and Hawkins [7], who found that patterns of distribution in relation to latitude differed among insect groups and that the generic richness of Ephemeroptera (mayflies) and Plecoptera (stoneflies) tended to be higher in temperate than tropical regions. Similarly, Pearson and Boyero [8] showed that species richness in these two insect orders tended to be higher at higher latitudes, although considerable variation was found on latitudinal gradients. Some of this variation is undoubtedly due to historical factors, including past speciation and extinction, as well as spatial and environmental processes [9]. Thus, community assembly (species sorting) can be mediated by the abiotic conditions and inter-species competition in the local environment, by regional factors that influence dispersal, and immigration to a local community [10,11], as well as by present and former land-use [12]. For example, in an extensive study of Brazilian streams, the community composition of Ephemeroptera was more strongly influenced by environmental (physico-chemical) factors than spatial processes [13].

It is also common for species richness of aquatic fauna to decline with increasing altitude, and the assemblage composition of stream invertebrates can also change profoundly, as found in the Himalayas of Nepal [14,15]. Although the factors determining altitudinal patterns are poorly known [16], temperature was clearly the best predictor of species richness for both plants and animals along a 3.7 km elevational gradient on Mount Kilimanjaro, Africa [17]. Ward [18] also implicated temperature as a prime determinant of the distribution, diversity, and abundance of aquatic insects along altitudinal gradients, a role that is hardly surprising given that it is a key factor affecting their growth, metabolism, reproduction, and emergence [19].

New Zealand is well suited for undertaking a latitudinal study as it is a narrow country whose three main islands extend over almost 13 degrees of latitude and whose mean air temperature declines by about 6 °C from north to south [20]. The country also possesses substantial mountain ranges and reasonable road access to about 1000 m a. s. l., which is close to the upper tree line, enabling altitudinal studies. A mean annual decline in temperature of −0.5 °C per 100 m has been reported from New Zealand [20], although the rate can be expected to vary depending on local topography and meteorological conditions [21].

The New Zealand mayfly fauna comprises 59 described species in 20 endemic genera and eight families, three of which are also endemic to the country [22,23,24]. The fauna shows close relationships with the mayflies of southern South America and Australia [25,26], which, like New Zealand, were parts of the ancient continent of Gondwana [27]. A number of the extant leptophlebiid genera also show affinities with New Caledonia [28], which, along with New Zealand, is an exposed fragment of the submerged Gondwanan landmass of Zealandia [29]. The distributions of New Zealand mayfly species have not been investigated systematically, but museum collections and general collecting, primarily for taxonomic purposes, suggest that more species are present in the northern and lowland streams than in the south of the country and at higher altitudes [28,30].

The aim of our study was to investigate the latitudinal and altitudinal distributions of New Zealand mayfly communities by means of an extensive and systematic nationwide survey incorporating light-trapping of imagos and subimagos and the collection of nymphs from streams. A subsidiary study was also undertaken at seven sites on Mount Taranaki, a prominent inactive volcano in the east of North Island, in which the confounding effect of latitude was factored out. We tested the hypothesis that species richness of mayflies in natural (unimpacted), predominantly forested, New Zealand streams would decline with increasing latitude and altitude, and that species turnover (beta diversity) would occur along both the latitudinal and elevational gradients in parallel with changes in species richness.

## 2. Materials and Methods

### 2.1. Study Sites

**Figure 1:**
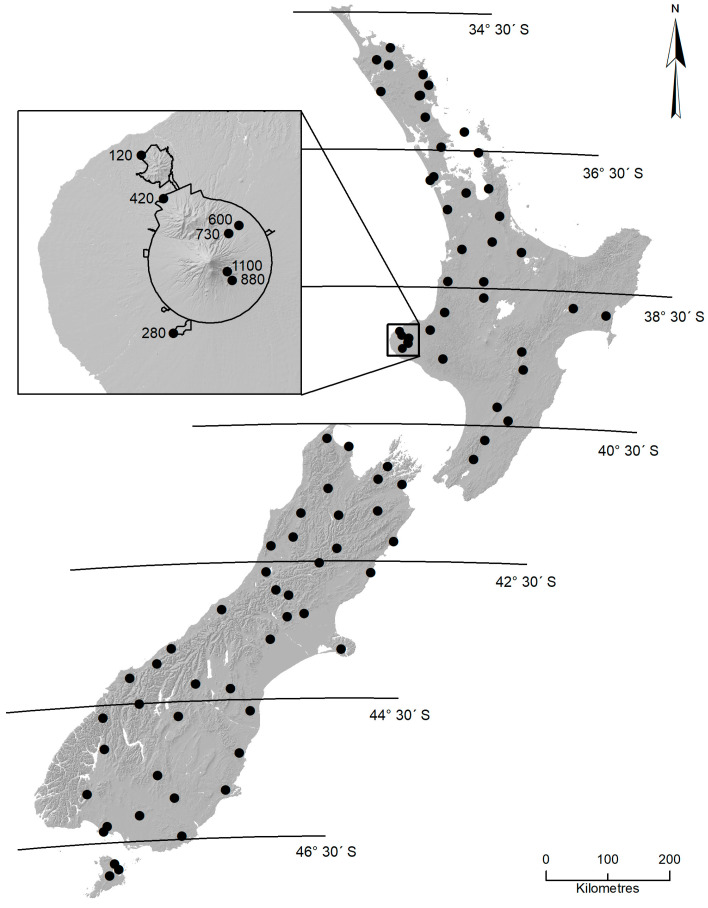
The 81 locations (243 sites) within New Zealand where sampling of mayfly communities was undertaken. Latitudinal lines delineate the zones used in analyses. The inset shows the locations and elevations of the seven sites used in the Mt Taranaki altitude study. The 600 m site was also sampled in the latitudinal survey. The black line in the inset indicates the boundary of Egmont National Park.

Mayflies were collected at 81 locations encompassing 13 degrees of latitude throughout New Zealand (Figure 1). At each location, sampling was undertaken at three well-separated sites (median distance apart 140 m) on second, third, or fourth order streams, giving a total of 243 sites.

To select the appropriate sampling locations, the country was divided into seven zones, each extending over 2 degrees of latitude. Between 11 and 15 locations were sampled within each zone, except in the southernmost Zone 7 (Stewart Island; 3 locations) whose land area is substantially smaller than that of the other zones. Specific locations conformed to the following criteria: (1) they were within predominantly unmodified landscapes (forest or subalpine environments); (2) they were as representative as possible of streams within their allocated zones, as indicated by the Freshwater Ecosystems of New Zealand (FENZ) geodatabase [31]; (3) they were broadly distributed within each zone and included a range of altitudes; and (4) they were accessible. Greater details on location selection, geographical coordinates for all 243 sites, and summaries of the physico-chemical data obtained from streams at the time of sampling have been reported by Pohe [32].

In addition to the nationwide latitudinal survey, sampling was carried out at seven sites representing an altitudinal gradient from 120 m to 1100 m a. s. l. on Mount Taranaki (Egmont), the second highest mountain in North Island (Figure 1). Mount Taranaki is a geologically young, quaternary volcano, whose activity is believed to have commenced about 200 thousand years ago [33]. The mountain forms most of Egmont National Park and encompasses natural protected ecosystems with minimal human-induced effects on water quality. Although altitudinal sampling could not be undertaken on a single stream, roads provided access to numerous similar-sized, stony stream sites surrounded by native podocarp–broadleaf forest at various elevations. Thus, except at the uppermost site (stream width 0.3 m), stream width at all sites ranges from 2.4 to 5.6 m. This design minimized the effects of differing catchment cover and land-use as well as latitude along the elevational gradient.

### 2.2. Survey Procedure

Fieldwork was carried out during the anticipated peak emergence period (austral summer) in three stages as follows: New Zealand’s North Island, November 2013–February 2014, New Zealand’s upper South Island, November 2014–February 2015, and New Zealand’s lower South Island (including Stewart Island), December 2015–January 2016. At all the sites, in each location, a light trap was set on a single night and nymphs were collected from a wide range of stream habitats by kick- and sweep-sampling, disturbing stones, wood, and leaf packs. Habitat variables were also measured (see Section 2.3 below). On six occasions, overnight trapping was disrupted by unfavorable weather (rain, cold temperature, wind), resulting in limited catches. Results for these sampling events were omitted from the analyses, leaving the 81 sampling locations presented in Figure 1.

Light traps used in the study comprised four 8-watt ultraviolet fluorescent tubes positioned above 9 L white trays half-filled with water [34]. Biodegradable Surfax^®^ detergent (Jasol New Zealand Ltd., Auckland, New Zealand) (30 mL) was added to the water to reduce surface tension, thereby enhancing the retention of trapped insects. Lights were powered by two 12-volt batteries (7–9 a/h) run in parallel and activated by timing modules set to activate at sunset and deactivate four hours later, thereby encompassing the period when the flight activity of New Zealand mayflies was expected to be highest [35,36]. Traps were placed either on the stream banks, or on large boulders or logs in mid-stream. Light trapping was mostly undertaken when there was no rain, little wind, and the air temperature at dusk was >13 °C, since preliminary sampling indicated that mayfly flight declines at lower temperatures [32]. Mayfly nymphs were collected from each site by the same person (SRP) who hand-picked specimens from rocks and wood, swept a net (30 cm at base; 500 µm mesh) across the substrate within pools, through trailing vegetation and leaf packs, and kick-sampled cobble–pebble riffles and coarse gravel stream runs. One hour was allocated for benthic sampling at each location (20 min per site). All mayflies collected (nymphs, subimagos, and imagos) were picked from collections on-site and preserved individually in Axygen^®^ microtubes (Corning, Glendale, AZ, USA) filled with 95% ethanol. Individual storage ensured that body parts, particularly legs, were retained for morphological analyses and prevented cross-contamination between the specimens needed for molecular analyses.

Sites used for the altitudinal survey on Mount Taranaki were sampled in December 2015 and February 2016 in the same way as those included in the nationwide survey.

### 2.3. Measurement of Habitat Variables

At each stream site, six habitat variables were measured within a 100 m long reach at the time of sampling. Wetted stream width was measured at five points and average stone size was determined by measuring the long-axis width of 50 randomly selected particles using the Wolman pebble count procedure [37]. Percent canopy cover was measured close to the stream surface with a spherical crown densiometer (Convex Model-A; Forestry Suppliers Inc., Jackson, MS, USA); four readings were taken at each of the three points within reach and then averaged. Stream water conductivity at 25 °C, a general measure of water quality [38], was determined with a YSI Professional Plus meter fitted with a Quatro multi-parameter sensor (Yellow Springs, OH, USA). Water temperature was logged for 12 h overnight, using Hobo^®^ pendant data loggers (model UA-002-64, Onset Computer Corporation, Bourne, MA, USA), and the values obtained every 15 min were then averaged. Finally, stream channel stability was evaluated using the Pfankuch index [39], which incorporates 15 measures of the stream banks and beds; to ensure consistency in the assessments, all stability evaluations were made by the same person (SRP).

### 2.4. Identification of Mayflies

Specimens were examined in the laboratory using a Nikon^®^ stereomicroscope (Model SMZ800, Nikon Corporation, Tokyo, Japan) at magnifications of 10–63× and identified using the taxonomic keys of Phillips [40,41], Towns and Peters [28], Hitchings and Staniczek [42], and Winterbourn et al. [43], in combination with original taxonomic descriptions. A number of ‘difficult’ specimens, mainly female imagos and subimagos, were also examined by molecular methods (COI barcodes) to confirm species-level identifications that were deemed likely to influence species counts, i.e., when several species of a genus were suspected to be present at the same location but could not be positively identified by microscopy. Molecular methods were also used to validate the identities of specimens which showed unexpected distributional ranges, were of potential conservation interest, were generally rare, or were suspected to be cryptic species. Details of the molecular methods used are given by Pohe [32].

### 2.5. Statistical Analyses

Data obtained in the nationwide survey were used to examine the associations between species composition of the mayfly fauna, latitude, and altitude. Data from individual sites at each location were pooled so the data matrix used for analyses comprised the total number of species (imagos, subimagos, and nymphs) found at each of the 81 locations. Subsequently, locality data were pooled to enable inter-zonal comparisons to be made. Zone 7 (Stewart Island), which had only three sampling locations, was excluded from inter-zonal analyses, because, on theoretical grounds, one might expect to collect fewer rare species there than in the other zones where sampling intensity was much greater [44]. Multiple linear regression was used to test the combined effect of latitude and altitude on species richness throughout New Zealand, and their individual effects were tested with simple linear regression.

Estimates of species turnover (beta diversity) among latitudinal zones were obtained using Sørensen’s similarity index as recommended by Buckley and Jetz [9] and Astorga et al. [45] and was calculated as *D* = 2c/a + b, where ‘a’ and ‘b’ are the total numbers of species in each zone and ‘c’ is species in common between the two zones. Comparisons with altitude were also made by combining locations into six altitudinal bands (13–40, 50–77, 100–123, 160–303, 330–465 and 548–973 m a. s. l.), designated such that each contained similar numbers of sampling locations.

Non-metric multidimensional scaling (NMS) incorporating the Bray–Curtis distance measure was used to ordinate the invertebrate communities in taxonomic space for the stream locations across New Zealand (81 locations, 52 species). Presence–absence data were used, and the solution with the lowest stress in two dimensions was selected as only small reductions in stress were obtained with the additional axes. Monte Carlo simulations (250 runs) were used to test the statistical significance of the ordination. The Similarity Percentage (SIMPER) procedure [46] was then used to identify which invertebrate taxa were most influential in determining regional differences. NMS was run in PC-ORD version 6.22 [47], and SIMPER was calculated in PAST version 4.02 [48]. The two NMS axes were correlated with the six measured habitat variables using Spearman’s rank correlation (*r_s_*) because NMS is not a linear ordination method [47]. In the Mount Taranaki study, correlations among altitude, air temperature, and species richness were made with Pearson’s *r*.

## 3. Results

### 3.1. Distribution of Species

The number of individual mayflies collected at the 81 locations ranged from 21 to 505 (mean 223), while the number of species per location ranged from 3 to 24 (mean 12.3).

**Figure 2:**
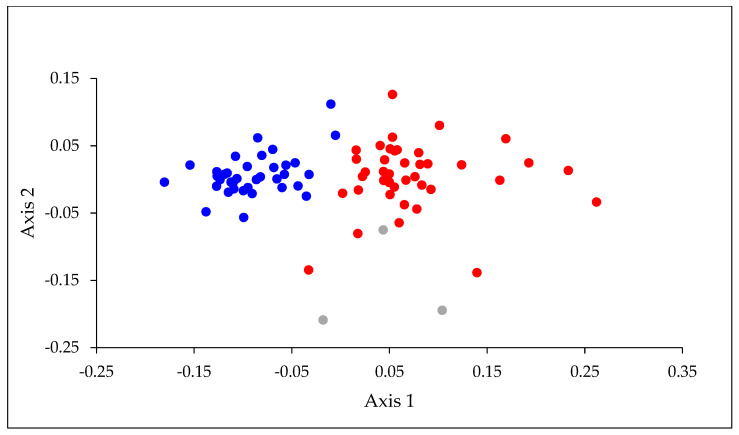
NMS ordination based on presence–absence data for mayfly species recorded at the 81 sampling locations. Stress = 15.3. The different colored symbols represent North Island (blue), South Island (red) and Stewart Island (grey) locations. Note: Stewart Island lies off the southern coast of South Island.

A total of 47 of the 59 mayfly species described from New Zealand, and five undescribed morphospecies, were collected during the nationwide survey (Table A1). Eleven species were widespread, occurring in all North and South Island zones, with 37 species being found in North Island and 34 in South Island, including southernmost Stewart Island. Of the 11 species most frequently encountered, all but two were recorded on both main islands (Table 1). The exceptions, *Acanthophlebia cruentata* and *Zephlebia dentata*, were confined to, but widespread, in North Island. An NMS ordination based on presence–absence data of the mayfly species separated almost all the North Island locations from those in the South Island (Figure 2).

SIMPER indicated that the species contributing most strongly to the separation of locations on the two islands were widely distributed on North Island and absent, or rarely found, on South Island (Table 2). When values for the six physical habitat variables, latitude, and altitude were correlated with axis 1 of the ordination, latitude had the strongest correlation (*r*_s_ = 0.79), followed by stream water temperature (−0.62), conductivity (−0.46), canopy cover (−0.41), and altitude (0.34). Axis 2 was most strongly correlated with canopy cover (−0.32) and the average size of stream bed substrata (Table 3). Our water temperature data, which were restricted to readings obtained on the days of sampling, had a range of 14.7 °C (6.7–21.4 °C) and, not surprisingly, were strongly correlated with latitude (*r_s_* = −0.63) and, to a lesser extent, altitude (*r_s_* = −0.46).

### 3.2. Relationships with Latitude and Altitude

**Figure 3:**
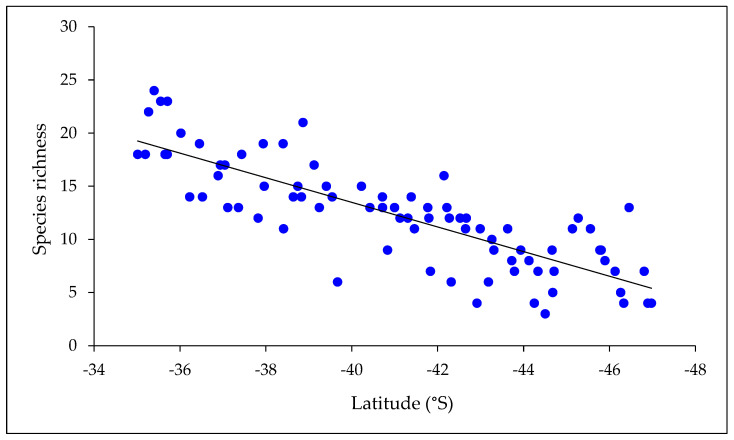
Mayfly species richness at all locations in relation to latitude. Richness declined significantly from north to south (*r*^2^ = 0.67; *p* < 0.001; *n* = 81).

**Figure 4:**
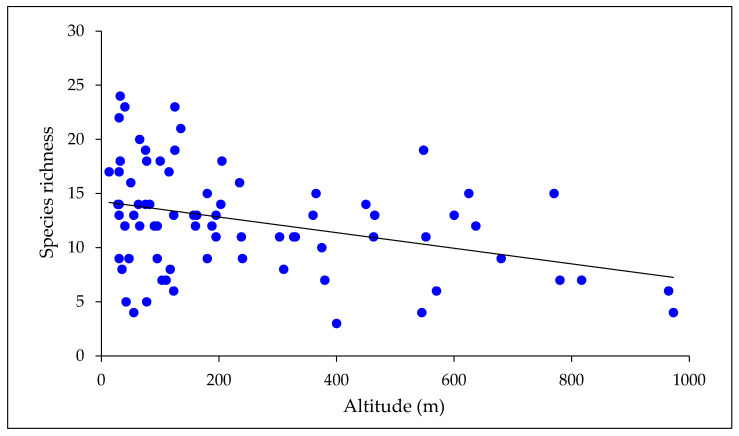
Mayfly species richness in relation to altitude. Richness declined significantly as altitude increased (*r*^2^ = 0.13; *p* < 0.01; *n* = 81).

Species richness of mayflies declined with both latitude and altitude (Figure 3 and Figure 4). A multiple regression with latitude and altitude as independent variables was highly significant (*r*^2^ = 0.68, *p* < 0.001), but the individual *r*^2^ values for the two variables indicated that latitude had a stronger effect on species richness (*r*^2^ = 0.64 cf. 0.13). When considered on a zonal basis, similarity of New Zealand mayfly communities declined with distance from north to south (Table 4).

A weaker relationship between species richness and altitude is evident in Figure 4. The number of mayfly species found in the four lower altitude bands ranged from 40 to 43, whereas 32 and 31 species were taken in the two higher altitude bands. The wide range of richness values found at low altitudes reflects high numbers of the species present in the north of the country, where low-lying land predominates, and a smaller pool of species available to colonize the lowland streams in the south. Mayfly communities became increasingly dissimilar as altitude increased but the degree of change was less pronounced than observed with latitude. Thus, the values of Sørensen’s *D* ranged from 0.74 to 0.95 among altitudinal zones, cf. 0.44 to 0.92 among latitudinal zones.

### 3.3. Mount Taranaki Altitudinal Surveys

Our surveys on Mount Taranaki encompassed an altitudinal range of 980 metres and also represented a temperature gradient (Table 5). Most of the mayflies collected were widely distributed species or had broad distributions in North Island [32]. Species richness declined significantly with altitude in both December and February and, of the 21 species found, 15 were taken at 120 m a. s. l. but only 5 were obtained at 1100 m a. s. l. (Table 5 and Table A2). *Deleatidium fumosum* was the only species recorded at all seven sites. In contrast, *Arachnocolus phillipsi* and *Deleatidium angustum* were only recorded at the lowest altitudinal site, whereas *D. magnum* and *Oniscigaster distans* were recorded only at the three highest and two highest altitudinal sites, respectively. Faunal similarity, as indicated by Sørensen’s index, was moderately high (*D* = 0.71–0.88) among all pairs of sites up to 730 m a. s. l. but then declined (Table 5).

## 4. Discussion

We found that the species richness of the mayflies declined significantly from the north to the south in New Zealand, despite the gradient extending over only 13 degrees of latitude. A comparable relationship between latitude and species richness was also reported for Australia by Lake et al. [49], who sampled stones in geomorphologically similar streams using a standardized procedure and found more invertebrate species in the tropical than the temperate regions of the country. Nevertheless, the diversity of higher aquatic taxa, such as insect orders, can differ greatly among streams along tropical–temperate gradients in Australia and elsewhere because of their great range of physico-chemical, hydrological, and climatic conditions, characteristics that inevitably confound regional patterns of taxonomic richness [50].

The relationship we found between species richness and altitude in our nationwide dataset was weaker than that between richness and latitude. The number of mayfly species in forest streams of comparable size, physico-chemistry, and surrounding vegetation also declined noticeably at sites > 730 m a. s. l. on Mount Taranaki and therefore showed a comparable pattern to that which was found in the nationwide survey. Together, these results are consistent with those of studies elsewhere, which indicate that species richness of Ephemeroptera is negatively associated with altitude and that altitude-related variables such as temperature play important roles in structuring mayfly communities [51,52].

In addition to a decline in species richness, the turnover (beta diversity) of mayfly communities increased from north to south, reflecting differences in the latitudinal distributions of individual species. Thus, of the 53 species recorded, only 11 were widespread across both North and South Islands, but 17 were found only on North Island and 16 only on South Island. Furthermore, some species were confined to the northernmost zones of the country, while others occurred throughout North Island, and a suite of species were found only in two or three South Island zones. A pattern of steadily declining species richness from north to south within New Zealand has also been described for canopy trees [53], although in the far north, where mayfly diversity is high, species richness of trees was low. McGlone et al. [53] postulated that the small land area of the Northland–Auckland peninsula was the reason for the low tree diversity there. In contrast, the northernmost region of the country has very high numbers of regionally endemic insect taxa, terrestrial amphipods, and land snails [54,55,56], and the exceptional richness of mayfly species in Northland may reflect its historical role as a faunal refuge and site of evolution during the Pleistocene ice ages [57,58,59]. Subsequent dispersal from this refuge is perceived to have extended the distributions of mayfly species southward to various extents, but Cook Strait and its predecessor, the Manawatu Strait [60], may have acted as barriers to the dispersal of mayflies into South Island [61]. Hence, the latitudinal pattern of change in species distributions from the north to the south can be explained, at least in part.

*Deleatidium*, however, with 21 described species, 10 of which are restricted to the South Island, appears to be an important exception to the above scenario. Thus, Hitchings [61,62] postulated that the formation of the Southern Alps and other South Island mountain ranges in the Miocene–Pliocene, and periodic advances and retreats of glaciers in the Pleistocene, would have resulted in the formation and diversification of new habitat and may have favored the speciation of *Deleatidium*. A similar scenario has been proposed for New Zealand blackflies (Diptera: Simuliidae) [63] and stoneflies (Plecoptera) [64], aquatic insects that are more strongly represented in the South Island and do not show a decline in species richness from the north to the south [63,65]. Today, nymphs of many *Deleatidium* species are found in moderate to fast-flowing waters, including many unstable, physically disturbed streams and rivers that characterize the South Island mountains [66]. In contrast, the nymphs of mayflies in several other genera of Leptophlebiidae found predominantly in the north tend to occur in slow-flowing parts of forest streams at low altitudes, consistent with a northern evolutionary origin in a less-mountainous environment [28].

As is well known, local environmental factors and the availability of a suitable habitat have important roles in structuring running water communities [67]. A pertinent example is the extensive Brazilian study of Shimano et al. [13], which indicated that environmental factors, including stream width, had a more significant role than spatial factors in structuring mayfly communities. Similarly, recent studies of New Zealand macroinvertebrate communities inhabiting riffles in forest streams suggested that habitat diversity had stronger effects on invertebrate richness than regional- or landscape-level factors [11,45]. While we did not explicitly investigate the role of local environmental and habitat factors on the distribution of mayflies, significant relationships with temperature, stream width, canopy cover, and conductivity suggest they are contributing factors in addition to latitude in determining ephemeropteran community composition and richness in New Zealand. Our results also suggest that the declining gradient in species richness from the north to the south probably has historical origins, with the north of the country having acted as a major refuge and region of speciation during the Pleistocene. The increasing dissimilarity of the northern and southern mayfly communities not only reflects differences in subsequent southward dispersal of these species but the presence of South Island species, which evolved in its mountains.

Because of its focus on latitude and altitude, our study has the potential to provide a basis for assessing the effects of ongoing climate change on New Zealand’s stream-dwelling Ephemeroptera and for evaluating their conservation needs. Over the course of the last century, there has been an increased frequency of extreme weather events, including warmer annual temperatures and reduced alpine snowfall [68,69]. These changes and the retreat and loss of glaciers have altered stream flows and habitats and can be expected to continue to affect the distribution of mayflies and other stream fauna, especially in the mountains. With an increase in temperature, the distributions of some species may move to higher altitudes or into cooler waters further south, but inhabitants of extreme cold-water habitats below the faces of glaciers are likely to be vulnerable to extinction [2,70].

In our nationwide survey, we recorded range-restricted mayfly species in many parts of the country, including the South Island mountains where several alpine and sub-alpine species of *Deleatidium* are potentially at risk [62,70]. Similarly, on Mount Taranaki, *D. magnum* and *Oniscigaster distans* were only found at the highest sites. Although *D. magnum* had been reported from a number of sites in the South Island mountains, prior to our study, the only record of its presence on the North Island was from Mount Ruapehu on the central plateau and on Mount Taranaki [32]. Its primarily South Island distribution, and apparent restriction to high mountain regions in North Island, suggest *D. magnum* is particularly vulnerable to future climate warming. *Oniscigaster distans* is also primarily a South Island species, although more widely distributed there than *D. magnum* [32,57]. However, in the North Island, it also appears to be uncommon and, of only 10 distribution records, 4 were obtained in the present study. With one exception, these records are all from mountainous regions [32] where stream water can be expected to be cooler at higher than at lower elevations. In contrast to the high-altitude and South Island species, mayflies currently confined to the warmer waters in northern New Zealand could also be at risk of at least local extinction if elevated water temperatures induce physiological stress, potentially leading to reductions in the size and extent of populations. The distribution of Ephemeroptera within New Zealand has undoubtedly changed markedly over many millennia as the geomorphology and climate of the country has evolved [61]. Our snapshot of their present distribution provides a baseline for the subsequent evaluations in a time of rapid climate change.

## Figures and Tables

**Table 1:** The 11 mayfly species most frequently encountered in the nationwide survey. All 11 species were found in both North and South Islands, except *A. cruentata* and *Z. dentata* ^1^ which were present in North Island only.

Species	Locations (%)
*Coloburiscus humeralis*	88
*Ameletopsis perscitus*	70
*Neozephlebia scita*	69
*Deleatidium myzobranchia*	62
*Deleatidium fumosum*	60
*Nesameletus ornatus*	57
*Zephlebia spectabilis*	51
*Austroclima sepia*	47
*Zephlebia versicolor*	46
*Acanthophlebia cruentata*	41
*Zephlebia dentata*	41

^1^ Mayflies from South Island previously recorded as *Z. dentata* are believed to be an undescribed species [32].

**Table 2:** The six mayfly species contributing most strongly to the separation of the North and South Island locations in the NMS ordination. The average dissimilarity value shows the mean percentage contribution of each species to the dissimilarity between North and South Islands as indicated by SIMPER.

Species	Average	Numbers of Locations Present (%)	
	Dissimilarity	North Island	South Island
*Acanthophlebia cruentata*	3.73	91.7	0
*Zephlebia dentata*	3.66	91.7	0
*Zephlebia versicolor*	3.25	88.9	11.9
*Zephlebia borealis*	3.03	77.8	0
*Austroclima sepia*	2.96	83.3	14.3
*Ichthybotus hudsoni*	2.79	72.2	0

**Table 3:** Spearman’s rank correlations of NMS axes 1 and 2 with altitude, latitude, and six habitat variables. * *p* < 0.01, ** *p* < 0.001, *** *p* < 0.0001.

	Altitude	Latitude	Stream Width	Canopy Cover	Stone Size	Pfankuch Index	Conductivity	Water Temp
Axis 1	0.34 *	0.79 ***	0.21	−0.41 **	0.28	−0.02	−0.46 ***	−0.62 ***
Axis 2	0.29 *	−0.20	0.29 *	−0.34 *	0.32 *	−0.16	−0.10	0.21

**Table 4:** Similarity of the mayfly fauna among latitudinal zones as indicated by Sørensen’s index. Each zone encompasses 2 degrees of latitude; Zone 1 being most northerly.

Zones	1	2	3	4	5	6
1	1.00	0.89	0.93	0.65	0.52	0.48
2		1.00	0.87	0.63	0.49	0.45
3			1.00	0.69	0.56	0.53
4				1.00	0.80	0.76
5					1.00	0.83
6						1.00

**Table 5:** Mean air temperature during light-trapping and numbers of mayfly species taken at seven sampling sites on Mount Taranaki in December 2015 and February 2016. Similarity of the total mayfly assemblages at each site to the fauna at the 120 m site is indicated by Sørensen’s *D*. Pearson correlations (*r*) and associated levels of significance (*p*) between air temperatures and altitude, and species richness and altitude are given in the right-hand columns.

	Altitude (m a. s. l.)								
	120	280	420	600	730	880	1100	*r*	*p*
Mean air temperature (°C)									
December	14.0	11.8	12.6	12.0	11.1	8.9	8.0	−0.94	0.002
February	18.1	17.7	16.5	15.2	14.7	15.7	13.7	−0.93	0.003
Number of species									
December	10	7	11	11	10	5	2	−0.67	0.11
February	13	11	13	8	9	8	4	−0.91	0.004
Total	15	12	13	13	12	8	5	−0.89	0.007
Similarity (*D*) cf. 120 m site	-	0.74	0.71	0.86	0.74	0.35	0.10		

## Data Availability

The raw data on which this work is based are available in the thesis Pohe, S.R. 2019. Macroecology of New Zealand Ephemeroptera. Unpublished PhD thesis, University of Canterbury, Christchurch, New Zealand. 226p. https://dx.doi.org/10.26021/7728.

## Appendix A

**Table A1:** Mayfly species collected in the nationwide survey of 81 locations throughout New Zealand and the latitudinal zone in which they were recorded. Species with informal names in inverted commas are undescribed morphospecies. The higher classification is that of Ogden and Whiting [71].

Species	Zones						
	1	2	3	4	5	6	7
Suborder Furcatergalia							
Family Ichthybotidae							
*Ichthybotus bicolor* Tillyard, 1923				x	x	x	
*Ichthybotus hudsoni* (McLachlan, 1894)	x	x	x				
Family Leptophlebiidae							
*Acanthophlebia cruentata* (Hudson, 1904)	x	x	x	x			
*Arachnocolus phillipsi* Towns & Peters, 1979	x	x	x				
*Atalophlebioides cromwelli* (Phillips, 1930)	x	x	x	x	x	x	
*Austroclima jollyae* Towns & Peters, 1979	x	x	x	x	x	x	
*Austroclima sepia* (Phillips, 1930)	x	x	x	x	x	x	x
*Austronella planulata* (Towns, 1983)	x	x	x				
*Deleatidium acerbum* Hitchings & Hitchings, 2016				x			
*Deleatidium angustum* Towns & Peters, 1996	x	x	x	x			
*Deleatidium atricolor* Hitchings, 2009				x	x	x	
*Deleatidium autumnale* Phillips, 1930	x		x	x	x	x	
*Deleatidium cerinum* Phillips, 1930	x	x	x	x	x		
*Deleatidium cornutum* Towns & Peters, 1996					x		
*Deleatidium fumosum* Phillips, 1930	x	x	x	x	x	x	
*Deleatidium kiwa* Hitchings, 2010						x	x
*Deleatidium lillii* Eaton, 1899	x	x	x	x	x	x	
*Deleatidium magnum* Towns & Peters, 1996			x				
*Deleatidium myzobranchia* Phillips, 1930	x	x	x	x	x	x	x
*Deleatidium patricki* Hitchings, 2008						x	
*Deleatidium townsi* Hitchings, 2009				x	x	x	
*Deleatidium vernale* Phillips, 1930	x		x	x	x	x	
*Deleatidium wardorum* Hitchings, 2010				x	x	x	
*Deleatidium* “lyellensis”				x			
*Deleatidium* “rakiura”							x
*Isothraulus abditus* Towns & Peters, 1979	x	x	x				
*Mauiulus aquilus* Towns & Peters, 1996	x						
*Mauiulus luma* Towns & Peters, 1979	x	x	x				
*Neozephlebia scita* (Walker, 1853)	x	x	x	x	x	x	x
*Tepakia caligata* Towns & Peters, 1996	x		x				
*Zephlebia borealis* (Phillips, 1930)	x	x	x	x			
*Zephlebia dentata* (Eaton, 1871)	x	x	x	x			
*Zephlebia inconspicua* Towns, 1983	x	x					
*Zephlebia nebulosa* Towns & Peters, 1996	x		x				
*Zephlebia pirongia* Towns & Peters, 1996		x					
*Zephlebia spectabilis* Towns, 1983	x	x	x	x	x	x	x
*Zephlebia tuberculata* Towns & Peters, 1996	x	x	x				
*Zephlebia versicolor* (Eaton, 1899)	x	x	x	x			
*Zephlebia* “aff. *pirongia* sp. 1”	x						
*Zephlebia* “sp. 1”				x	x		
Suborder Pisciforma							
Family Ameletopsidae							
*Ameletopsis perscitus* (Eaton, 1899)	x	x	x	x	x	x	x
Family Nesameletidae							
*Nesameletus austrinus* Hitchings & Staniczek, 2003				x	x	x	
*Nesameletus flavitinctus* (Tillyard, 1923)	x	x	x	x	x		
*Nesameletus murihiku* Hitchings & Staniczek, 2003							x
*Nesameletus ornatus* (Eaton, 1883)	x	x	x	x	x	x	
*Nesameletus* “Fiordland”					x	x	
Family Oniscigastridae							
*Oniscigaster distans* Eaton, 1899			x	x	x	x	
*Oniscigaster wakefieldi* McLachlan, 1873	x	x	x	x		x	
Family Rallidentidae							
*Rallidens mcfarlanei* Penniket, 1966	x	x	x				
*Rallidens platydontis* Staniczek & Hitchings, 2014				x		x	
Family Siphlaenigmatidae							
*Siphlaenigma janae* Penniket, 1962	x	x	x		x		
Suborder Setisura							
Family Coloburiscidae							
*Coloburiscus humeralis* (Walker, 1853)	x	x	x	x	x	x	x

**Table A2:** Altitudes (metres a. s. l.) at which mayfly species were collected on Mount Taranaki.

Species	120	280	420	600	730	880	1100
*Acanthophlebia cruentata*	x	x	x	x	x		
*Ameletopsis perscitus*	x	x	x	x	x	x	
*Arachnocolus phillipsi*	x						
*Austroclima jollyae*		x					
*Austroclima sepia*	x			x	x		
*Coloburiscus humeralis*	x	x	x	x	x		
*Deleatidium angustum*	x						
*Deleatidium autumnale*			x			x	x
*Deleatidium fumosum*	x	x	x	x	x	x	x
*Deleatidium magnum*					x	x	x
*Deleatidium myzobranchia*	x	x	x	x	x	x	
*Ichthybotus hudsoni*	x		x				
*Neozephlebia scita*	x	x	x	x	x		
*Nesameletus flavitinctus*			x	x	x	x	x
*Nesameletus ornatus*	x			x			
*Oniscigaster distans*						x	x
*Rallidens mcfarlanei*			x				
*Zephlebia borealis*	x	x	x	x			
*Zephlebia dentata*	x	x		x	x		
*Zephlebia spectabilis*	x	x	x	x	x		
*Zephlebia versicolor*	x	x	x	x	x	x	
